# Electrochemical buckling microfabrication†
†Electronic supplementary information (ESI) available: Detailed fundamentals of CELT, ECBM experiments, FEM simulation and PL image. See DOI: 10.1039/c5sc02644j



**DOI:** 10.1039/c5sc02644j

**Published:** 2015-10-20

**Authors:** Jie Zhang, Bo-Ya Dong, Jingchun Jia, Lianhuan Han, Fangfang Wang, Chuan Liu, Zhong-Qun Tian, Zhao-Wu Tian, Dongdong Wang, Dongping Zhan

**Affiliations:** a State Key Laboratory of Physical Chemistry of Solid Surfaces , Department of Chemistry , College of Chemistry and Chemical Engineering , Xiamen University , Xiamen , 361005 , China . Email: dpzhan@xmu.edu.cn; b College of Architecture and Civil Engineering , Xiamen University , Xiamen , 361005 , China . Email: ddwang@xmu.edu.cn

## Abstract

Can isotropic wet chemical etching be controlled with a spatial resolution at the nanometer scale, especially, for the repetitive microfabrication of hierarchical 3D μ-nanostructures on the continuously curved surface of functional materials? We present an innovative wet chemical etching method called “electrochemical buckling microfabrication”: first, a constant contact force is applied to generate a hierarchical 3D μ-nanostructure on a mold electrode surface through a buckling effect; then, the etchant is electrogenerated on-site and confined close to the mold electrode surface; finally, the buckled hierarchical 3D μ-nanostructures are transferred onto the surface of a Ga_*x*_In_1–*x*_P coated GaAs wafer through WCE. The concave microlens, with a Fresnel structure, has an enhanced photoluminescence at 630 nm. Comparing with energy beam direct writing techniques and nanoimprint lithography, this method provides an electrochemical microfabrication pathway for the semiconductor industry, with low cost and high throughput.

## Abstract 2

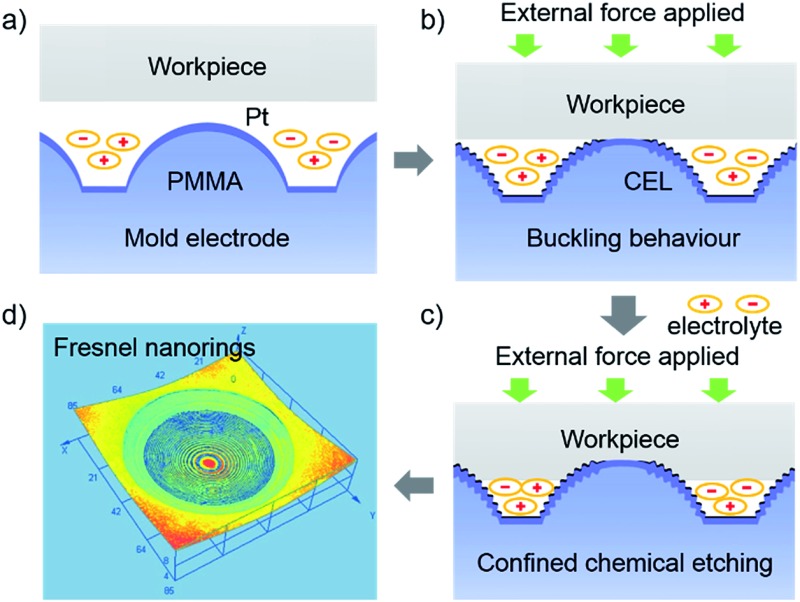
Isotropic wet chemical etching can be controlled with a spatial resolution at the nanometer scale, especially for the repetitive microfabrication of hierarchical 3D μ-nanostructures on the continuously curved surface of functional materials.

## Introduction

Microfabrication is a current topic and plays a crucial role in the industrial fabrication of devices, such as ultra large scale integrated circuits,^1^ precision optics,^2^ microelectromechanical systems,^3^ and miniaturized total analytical systems.^4,5^ Nevertheless, a great challenge remains to fabricate hierarchical three-dimensional micro- and nano-structures (3D μ-nanostructures) directly onto functional materials with a continuously curved surface, *e.g.*, the artificial compound eye in optics. Photolithography is difficult to do, due to the rectilinear propagation of light.^6^ Direct writing techniques based on an electron beam,^7^ ionic beam^8^ or laser beam^9,10^ are capable of fabricating 3D μ-nanostructures. However, the huge workload, high cost and serious surface damage hinder their applications in repetitive manufacturing.^11^ Besides the difficulty of mold preparation, nanoimprinting works only with thermoplastic or photocurable materials, which are usually not the functional materials aimed for.^12,13^ Thus, it is essential to develop new principles and methods to meet the increasing demands of hierarchical 3D μ-nanostructures in industrial microfabrication.

Wet chemical etching (WCE) was one of the first techniques introduced for microfabrications.^14,15^ Proceeding along the special crystal plane, nanostructures made by anisotropic WCE onto the surface of single crystalline materials are facet-dependent.^16,17^ The spatial resolution of isotropic WCE is ruined by it having the same etching rate in all directions.^18,19^ The common disadvantage of this method is that the process is uncontrollable. Either over-etching or under-etching would harm the product consistency. That's why, in the semiconductor industry, WCE is now underused as a wafer cleaning process.

How to control the WCE process? For years we have developed a confined etchant layer technique (CELT) to solve the problem:^20–24^ firstly, the etchant is generated on the surface of a mold electrode by an electrochemical reaction. Secondly, the diffusion distance of the etchant is confined to the micron or nanometer scale by a subsequent homogeneous reaction. Consequently, a confined etchant layer (CEL) is formed on the mold electrode surface (Fig. S1 in ESI†). When the CEL is brought into contact with the workpiece by a nanomanipulation system, WCE begins. Most importantly, the WCE process will stop automatically if the workpiece material is removed and, therefore, separated from the CEL. In the case of the Ga_*x*_In_1–*x*_P substrate (for GaAs it has been well discussed elsewhere^14^), the CELT strategies are formulated as follows:

The generating reaction of the etchant:

1 18Br^–^ → 9Br_2_ + 18e^–^

The confining reaction of the etchant:

2 5Br_2_ + RSSR + 6H_2_O → 2RSO_3_H + 10Br^–^ + 10H^+^

The WCE reaction:

3 4Br_2_ + Ga_*x*_In_1–*x*_P + 4H_2_O → Ga_*x*_^3+^ + PO_4_^3–^ + In_1–*x*_^3+^ + 8H^+^ + 8Br^–^

where RSSR is l-cystine. If the concentration of l-cystine is much higher than that of the etchant precursor, the thickness of the CEL can be estimated simply by the following equation:^25^

4 *μ* = (*D*/*K*_s_)^½^

where *μ* is the thickness of the CEL, *D* is the diffusion coefficient of the etchant, and *K*_s_ is the quasi-first-order rate constant of the confining reaction. If *D* and *K*_s_ were 10^–5^ cm^2^ s^–1^ and 10^9^ s^–1^, the thickness of the CEL would be ∼1 nm. That means, theoretically, CELT is a very precise microfabrication method. In this way, the WCE process is well controlled at the micron or nanometer scale.

**Fig. 1:**
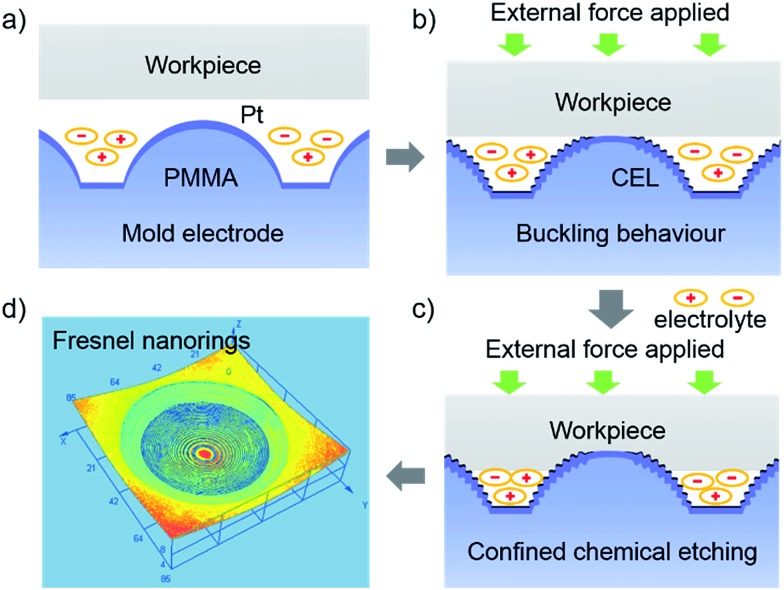
Schematic illustration of the ECBM process. (a) The workpiece approaches the PMMA/Pt working electrode with a convex microlens array. (b) When the workpiece contacts the PMMA/Pt working electrode, a constant contact force is applied to produce the hierarchical Fresnel nanostructure on the surface of the microlens through buckling. Then the PMMA/Pt working electrode is biased at 1.0 V (*vs.* SCE) to generate the confined etchant layer. (c) With the confined wet chemical etching occurring, the concave microlenses, with hierarchical Fresnel nanostructures, are transferred to the workpiece. (d) The hierarchical 3D μ-nanostructures obtained through ECBM.

How to generate hierarchical 3D μ-nanostructures and transfer them onto functional materials? Buckling is a natural phenomenon that denotes a geometric instability for a given structure under compression or shear stresses.^26^ Recently, the buckling effect has aroused extensive interest in the fabrication of 3D μ-nanostructures.^27–35^ By introducing elastic buckling into CELT, as depicted in Fig. 1, we develop a method called electrochemical buckling microfabrication (ECBM). When a constant contact force is applied between the workpiece and mold electrode, orderly hierarchical 3D μ-nanostructures are formed on the surface of the convex microlens through the buckling effect due to the difference in the elastic modulus between the rigid platinum (Pt) film and the plastic polymethylmethacrylate (PMMA) substrate (Fig. S2†). Then, the buckled 3D μ-nanostructures are transferred onto the surface of the Ga_*x*_In_1–*x*_P thin film coated GaAs wafer by CELT.

## Results and discussion

### Fabrication of microlens with hierarchical Fresnel nanorings

**Fig. 2:**
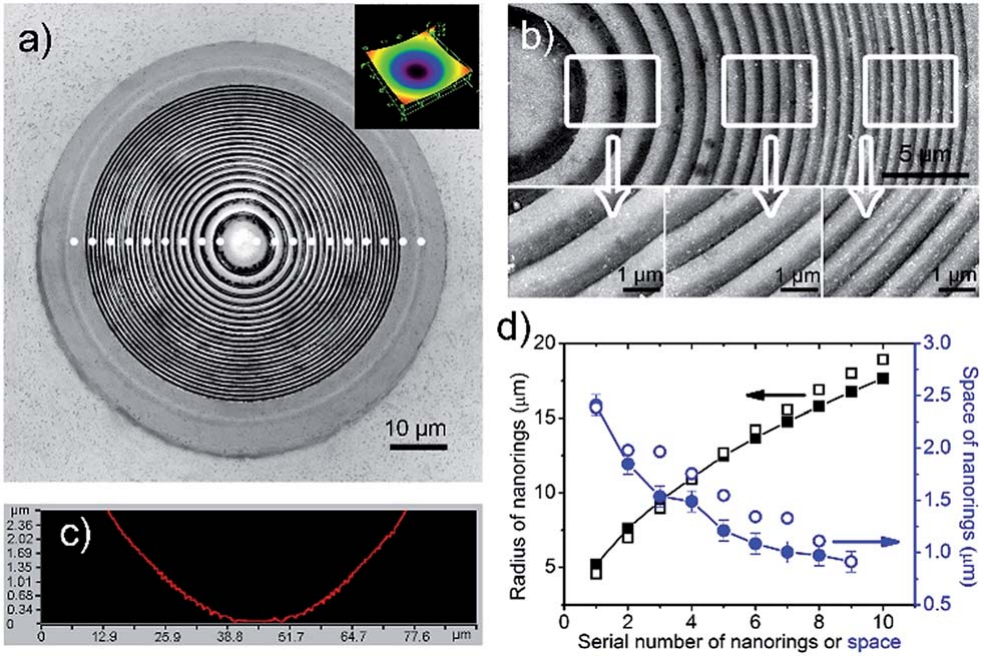
Hierarchical Fresnel nanostructures fabricated through ECBM on the Ga_*x*_In_1–*x*_P workpiece with a 20 mN contact force. (a) Confocal laser microscope image showing 23 concentric nanorings. Inset shows its 3D image. (b) High-resolution SEM images of the nanorings. (c) Topography profile of (a). (d) The change in radius and space as a function of the first ten nanorings from the centre outwards. Solid symbols represent the experimentally determined radii and spaces. Open symbols represents the FEM simulated results.

In ECBM, the working electrolyte is an aqueous solution containing 0.1 M KBr, 0.1 M l-cystine and 0.5 M H_2_SO_4_. With a Pt counter electrode, a saturated calomel reference electrode (SCE) and an applied potential of 1.0 V at the mold electrode (working electrode), bromine (Br_2_) is generated to start the WCE process. Fig. 2a and b show the confocal laser microscopic images of the hierarchical Fresnel structure (*i.e.*, concentric nanorings) on the concave microlens, obtained with a constant contact force of 20 mN. The nanoring at the center has a height of 50 nm and also decays from the center outwards (Fig. 2c and S3†). From Fig. 2d, it is observed that, from the center outwards, the radius of the first ten nanorings increases gradually from 5.2 μm to 17.7 μm, while the gap between neighboring nanorings decreases from 2.4 μm to 900 nm. In the control experiment, when the workpiece is not in contact with the mold electrode, concave microlenses without nanorings are fabricated (Fig. S4†). The buckled nanorings on the mold electrode disappear when the contact force is released. This elastic buckling behavior can avoid tool wear and make ECBM work repetitively; note that tool wear is a serious problem for nanoimprint lithography in mass production.

**Fig. 3:**
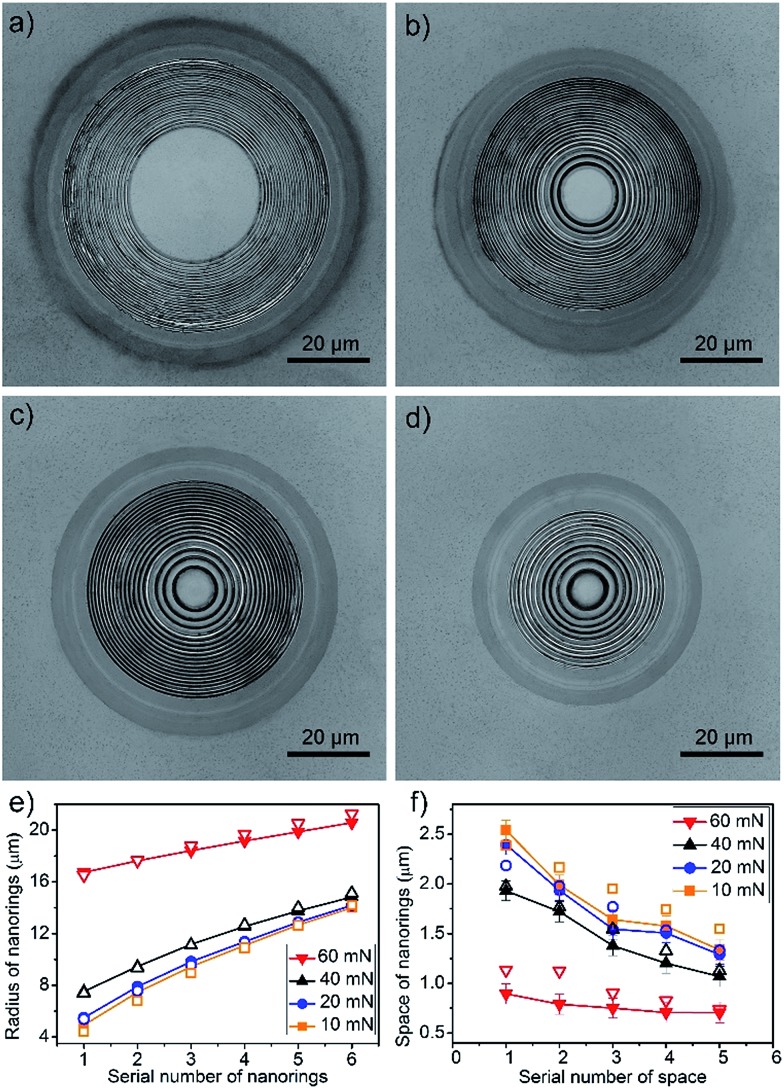
Confocal laser microscopic images of the hierarchical Fresnel nanostructures fabricated by ECBM with different contact forces. (a) 60 mN. (b) 40 mN. (c) 20 mN. (d) 10 mN. The change in radius (e) and space (f) as a function of the first six nanorings from the centre outwards at different contact forces. Solid symbols represent the experimentally determined radii and spaces. Open symbols represent the FEM simulated results.

The hierarchical Fresnel structure on the concave microlens can be tuned by the applied contact force. Fig. 3 shows the Fresnel structures fabricated with different contact forces of 60 mN, 40 mN, 20 mN and 10 mN. The radius of the first nanoring increases from 4.9 μm to 16.7 μm with the increasing contact force (Fig. 3e). Because the mold electrode is compressed tightly on the workpiece, buckling doesn't occur in the central contact area. The larger the applied force, the larger the central area obtained. Moreover, the Fresnel structure becomes denser when the applied force increases. The gap between the first two nanorings decreases from 2.5 μm to 900 nm when the contact force changes from 10 mN to 60 mN. Although the gap between neighbor nanorings decreases from the center outwards, they tend to be more uniform with increasing contact force (Fig. 3f and S5†).

### Simulation of the buckling behaviour on the mold electrode

**Fig. 4:**
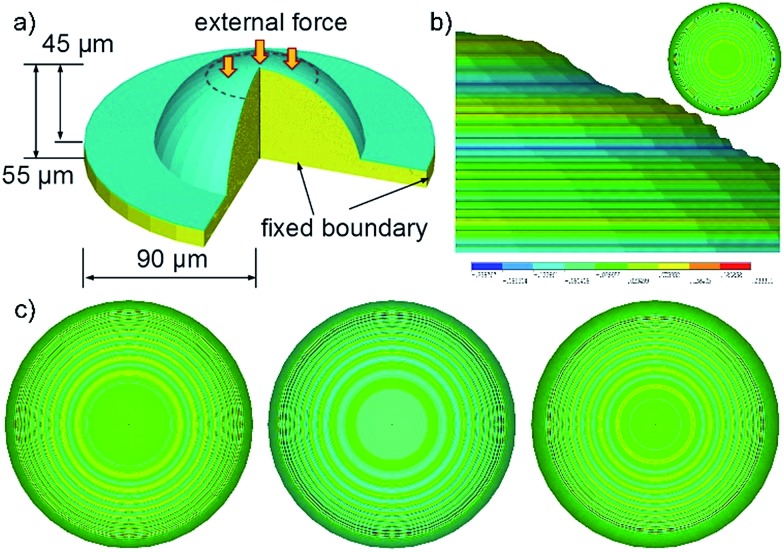
Finite element simulation results of the buckling behavior at a convex microlens. (a) The geometric properties and boundary conditions of the established model. (b) The side view of the buckling patterns at 20 mN contact force. Hierarchical nanorings are formed on the surface of the microlens. The inset shows the top view of the buckled microlens. (c) Top view of the nanorings buckled at different contact forces: 60 mN, 40 mN and 10 mN from left to right.

Experimentally, we obtain buckled 3D μ-nanostructures on the continuously curved surface and transfer them onto functional materials. Now we discuss the buckling effect with the finite element method (FEM) using the high-fidelity ANSYS finite element package.^36^ Since the mold electrode is composed of a PMMA convex microlens array coated with a thin Pt film, the geometry and mechanical properties used in the simulation are shown in Fig. 4a. The Young's modulus of the Pt film and PMMA substrate is 168 GPa and 20 GPa (see S9†), while the Poisson's ratios are 0.38 and 0.4, respectively. Both the Pt film and the PMMA microlens are modeled as elastic materials. A Saint-Venant type of constitutive equation is employed to relate the stress and strain tensors as follows:5
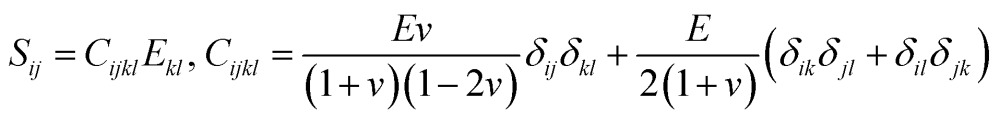
where *C*_*ijkl*_ is the elastic tensor, *δ*_*ij*_ is the Kronecker delta tensor, and *E* and *v* represent the Young's modulus and Poisson's ratio. In simulations, the thickness of the Pt film on the PMMA convex microlens is non-uniform (changing gradually from 248 nm at the top to 30 nm at the bottom, see S10†), which often happens when sputter coating on 3D structures. For more details of FEM simulations, please check Section 6.2 in the ESI.†

When a contact force is applied to the mold electrode, the Pt film suffers compressive stress and passes it onto the PMMA substrate. If the compressive stress exceeds the critical buckling stress, the equilibrium of the system is interrupted and the buckled 3D μ-nanostructures appear, due to an initial imperfection or small perturbation. The buckling instability leads to the generation of the hierarchical Fresnel structure on the continuously curved convex microlens. Fig. 4b shows the buckling behaviour of the mold electrode with a constant contact force of 20 mN. The gap between nanorings decreases from the center outwards, which is mostly attributed to the non-uniform thickness of Pt film and the distribution of stress states on the convex microlens. The thicker film on the top of the convex microlens has a larger bending rigidity and yields sparser nanorings.^26,37^ On the contrary, the thinner film at the bottom results in denser nanorings. Thus, the gap between nanorings is related to the thickness distribution of Pt film on the PMMA microlens, which also provides a way of tuning the buckled 3D μ-nanostructures.

Simulations demonstrate that the buckled 3D μ-nanostructures are also influenced by the applied contact force. As shown in Fig. 4c, the gap between neighbour nanorings decreases with the increased compression. In the conventional case of a planar film, a larger compressive stress produces a smaller wavelength of buckling.^38^ However, for the Pt film coated on the PMMA convex microlens discussed herein, the compressive stress in the Pt film reduces gradually from top to bottom (Fig. S6†). The increasing contact force leads to a larger compressive stress, which results in denser buckling of the nanorings (Section 6.3 in ESI†). FEM simulations show that the distribution of the bending rigidity and compressive stress of the Pt film coated on the convex microlens has an opposite effect on the density of the nanorings, *i.e.*, the nanorings tend to become more uniform with the increasing contact force (Fig. 4c). For more discussion, please check Section 6.3 in the ESI.†

### Photoluminescence of the microlens

**Fig. 5:**
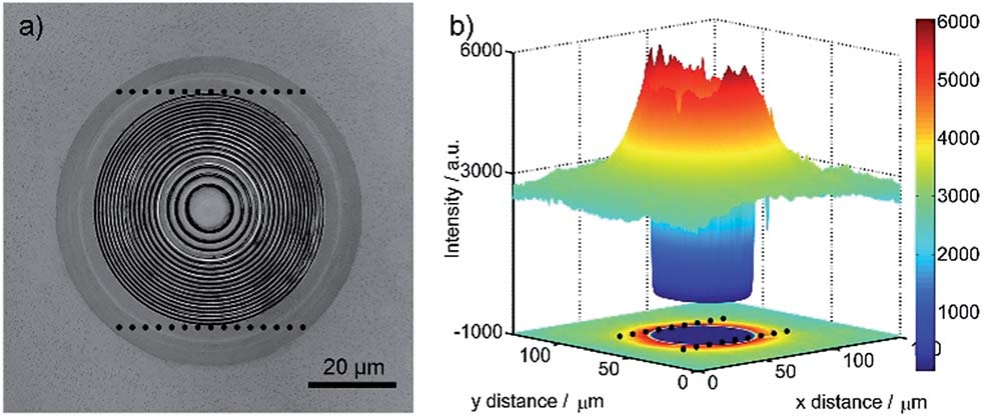
The photoluminescence of the concave microlens with hierarchically concentric nanorings: (a) the confocal laser image of a single microlens and (b) the corresponding photoluminescence image at 630 nm wavelength.

Since the workpiece is a GaAs wafer coated with a thin Ga_*x*_In_1–*x*_P layer with a thickness of 5 μm, the outside area of the obtained concave microlens is Ga_*x*_In_1–*x*_P and the central area is GaAs, with a transition region between them. In fact, each concave microlens with hierarchical Fresnel nanostructures is a semiconductor light-emitting diode (LED) with a quantum-well structure. Besides the photoelectric properties, the microlens has a structure-dependent, enhanced photoluminescence (PL) property. When the microlens shown in Fig. 5a is illuminated by a 532 nm-wavelength laser, a gradient and circular PL belt can be detected with a single-wavelength at 630 nm. The PL intensity is enhanced in the narrow outside area and quenched in the central area, *i.e.*, the incident light is sufficiently trapped by the hierarchical Fresnel nanostructures on the concave microlens. The results illustrate that ECBM has prospective applications in semiconductor LEDs and optical microdevices. The PL images of single concave microlenses with hierarchical Fresnel nanostructures obtained using different contact forces are shown in Fig. S9.†

## Conclusions

In conclusion, an electrochemical buckling microfabrication method is developed by introducing physical modulations into confined wet chemical etching: elastic buckling, a physical self-assembly behavior, is adopted to generate hierarchical 3D μ-nanostructures on a continuously curved surface, which are then transferred onto the functional materials by a confined etchant layer technique. The fabricated concave microlens with hierarchical Fresnel nanostructures has an excellent structure-dependent photoluminescence. Besides the hierarchical nanorings, parallel nano-grooves are also fabricated on the surface of the concave hemicylinder (Fig. S10†). Compared with nanoimprint and energy beam direct-writing techniques, ECBM provides a controllable, highly-efficient and low-cost electrochemical approach for the fabrication of semiconductor devices with hierarchical 3D μ-nanostructures.

## Experimental section

### Sample preparation

All the chemicals used (KBr, H_2_SO_4_, l-cystine) are analytical grade or better and provided by Sinopharm Co., China. PMMA is purchased from Mitsubishi Rayon Co., Japan. The Ga_*x*_In_1–*x*_P wafers are a gift from Prof. Jingqiu Liang at the Changchun Institute of Optics, Fine Mechanics and Physics, Chinese Academy of Science. All solutions are prepared with deionized water (18.2 MΩ, Milli-Q, Millipore Corp.).

### Fabrication of the mold electrode

The concave microlens array on the titanium plate (diameter: 110 μm, height: 45 μm) is imprinted onto the PMMA substrate by a hot embossing technique. After cooling, the PMMA substrate is immersed in a solution of 50 g L^–1^ Na_3_PO_4_, 25 g L^–1^ Na_2_CO_3_ and 20 g L^–1^ NaOH at 85 °C for 2.5 h to remove the oil. Thin films of titanium (thickness: 5 nm) and platinum are sputtered onto the PMMA mode successively to produce the conductive mold electrode.

### Electrochemical buckling microfabrication

A home-made instrument (Fig. S1d†) is used to perform the ECBM. The mold electrode is fixed and is moved towards the workpiece using a nano-manipulator. A force sensor is used to monitor the contact force between the mold electrode and the workpiece. After the buckled 3D μ-nanostructures are generated on the mold electrode, an electrochemical workstation (CHI 660D, CHI Instrument Co., USA) is used to perform the CELT process. A Pt wire and a SCE are used as the counter electrode and reference electrode. The mold electrode is biased at 1.0 V to generate the etchant bromine. l-Cystine is added to form CEL. During the etching process, the contact force is kept constant.

### Characterizations and measurements

Confocal laser microscopy (Olympus 4000, Olympus Co., Japan), scanning electron microscopy (Hitachi S-4800, Hitachi High-Technologies Co., Japan), and atomic force microscopy (Nanoscope III scanning probe microscope, Digital Instrument Co., USA) are employed to characterize the hierarchical 3D μ-nanostructures. Laser Raman microscopy (Nanophoton laser Raman microscope, RAMAN-11) in a fast line scanning mode with a 50× objective (NA = 0.45), 300 lines per mm grating and 532 nm-wavelength laser (power: 0.029 mW) is employed to characterize the photoluminescence of the nanorings.
