# Supplementary material for: Electrochemical buckling microfabrication
Source: Chem Sci. 2015 Oct 20;7(1):697–701. doi: 10.1039/c5sc02644j (PMC5523117; doi:10.1039/c5sc02644j)
Supplement: Supplementary file 1 [file SC-007-C5SC02644J-s001.pdf]

Electronic Supplementary Material (ESI) for Chemical Science.
This journal is © The Royal Society of Chemistry 2015

Supplementary Information

Electrochemical Buckling Microfabrication

Jie Zhang,[a] Bo-Ya Dong,[b] Jingchun Jia,[a] Lianhuan Han,[a] Fangfang Wang,[a] Chuan Liu,[a] Zhong-Qun Tian,[a] Zhao-Wu Tian,[a] Dongdong Wang[b] and Dongping Zhan*[a]*

[a]State Key Laboratory of Physical Chemistry of Solid Surfaces, and Department of Chemistry, College of Chemistry and Chemical Engineering, Xiamen University, Xiamen, 361005, China. E-mail: dpzhan@xmu.edu.cn
[b]College of Architecture and Civil Engineering, Xiamen University, Xiamen, 361005, China. E-mail: ddwang@xmu.edu.cn

Content

1. Fundamentals of CELT
2. Topographic characterization of mold electrode
3. AFM characterization of hierarchically concentric nanorings
4. Control experiments without contact force
5. Tuning the hierarchical nanorings through contact force
6. The stress and platinum film thickness dependent hierarchical nanorings
   6.1 FEM simulation of buckling behavior
   6.2 The reversible buckling process
   6.3 Details of FEM simulation
7. The photoluminescence mapping of the hierarchical nanorings
8. Tuning the hierarchical nanogrooves through contact force
9. The characterization of Young's modulus of PMMA substrate
10. The non-uniform distribution of Pt film on PMMA mold
11. AFM characterization of the amplitude of concentric nanorings

1. Fundamentals of CELT

Confined etchant layer technique (CELT) is a chemical etching technique induced by in-situ photo/electrochemistry.[1] In principle, there are three chemical strategies of CELT for the fabrication of complex 3D micro- and nano-structures:

(1) Generating the etchant at working electrode:

$$R \rightarrow O + ne \text{ or } R + h\upsilon \rightarrow O\ (+ ne) \qquad (S1)$$

where R is the precursor of etchant, O is the etchant species. Actually, the working electrode used in CELT is also the mold for micro-nano fabrication. The precursor of etchant is oxidized photo/electrochemically to generate etchant on the surface of the mold electrode. Because of the free diffusion of etchant in the working solution, the resolution of chemical etching process is difficult to control (Figure S1a). Thus, a scavenger which can react with etchant is adopted to confine the diffusion of etchant.

(2) Confining the diffusion layer of etchant to micro- or nanometer scale:

$$O + S \rightarrow R + Y \text{ or } O \rightarrow Y \qquad (S2)$$

where S is the scavenger and Y is the product of S reacts with O or the decay product if O is a radical, Due to the subsequent chemical reaction ($E_rC_i^{'}$) between the etchant and scavenger, the thickness of confined etchant layer will become thinner (Figure S1b). The electrochemical behavior of the E_rC_i' reaction can be seen in Figure S1c. Without L-cystine (scavenger), the redox reaction of Br^-/Br_2 is reversible. The peak potential difference of the oxidation and reduction process (ΔE_p) is about 60 mV. With the existence of L-cystine, the redox behavior becomes irreversible since the electro-generated Br_2 is consumed quickly by the scavenger.[2] The inset Figure S1c shows the cyclic voltammogram of mold electrode with convex microlens array. Through the scavenging reaction, the thickness of CEL can be confined to micro- and nanometer scale, which determines the fabrication precision of CELT.

(3) Micro-nano fabrication through chemical etching:

$$O + M \rightarrow R + P \qquad (S3)$$

When the workpiece (M) approaches to the mold electrode, vice versa, until the CEL contacts with the surface of workpiece, chemical etching will occur. Feeding the mold electrode, complementary micro-nano structures will be obtained on workpiece. Since the thickness of

CEL is confined to micro-nanometer scale, the CEL will keep the shape of the mold electrode. The fabrication precision of CELT is determined by the thickness of CEL. Figure S1d shows schematic of home-made instrument used for CELT. The electrochemical system and 3D nanomanipulation system are the two main parts in the instrument.

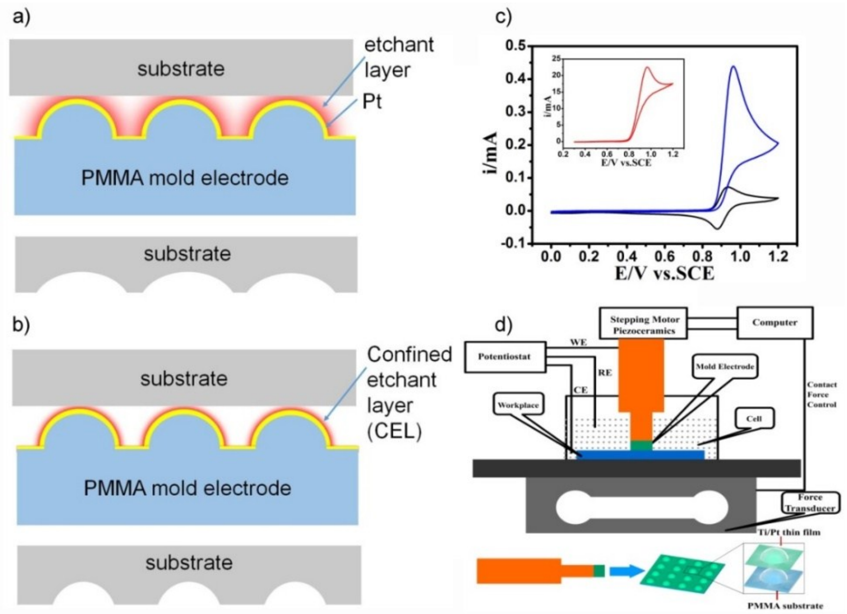

Figure S1. The schematic of CELT. a), b) Schematic showing the chemical etching processes without and with CELT, respectively. c) Cyclic voltammograms of Pt disk electrode (diameter 2 mm) in the solution of 8.3 mM KBr + 0.5 M H_2SO_4 (black line) and 8.3 mM KBr +8.3 mM L-cystine + 0.5 M H_2SO_4 (blue line). The inset (red line) shows the cyclic voltammograms of mold electrode with convex microlens array in the solution of 0.1 M KBr + 0.1 M L-cystine + 0.5 M H_2SO_4. d) Schematic diagram of the home-made instrument of CELT.

2. Topographic characterization of mold electrode

The PMMA mold electrode covered with Pt film fabricated by hot embossing technique is characterized by confocal laser microscope (Figure S2a). The convex microlens with 55 μm radius and 45 μm height is used in the electrochemical buckling microfabrication and FEM simulation (Figure S2b and Figure S2c).

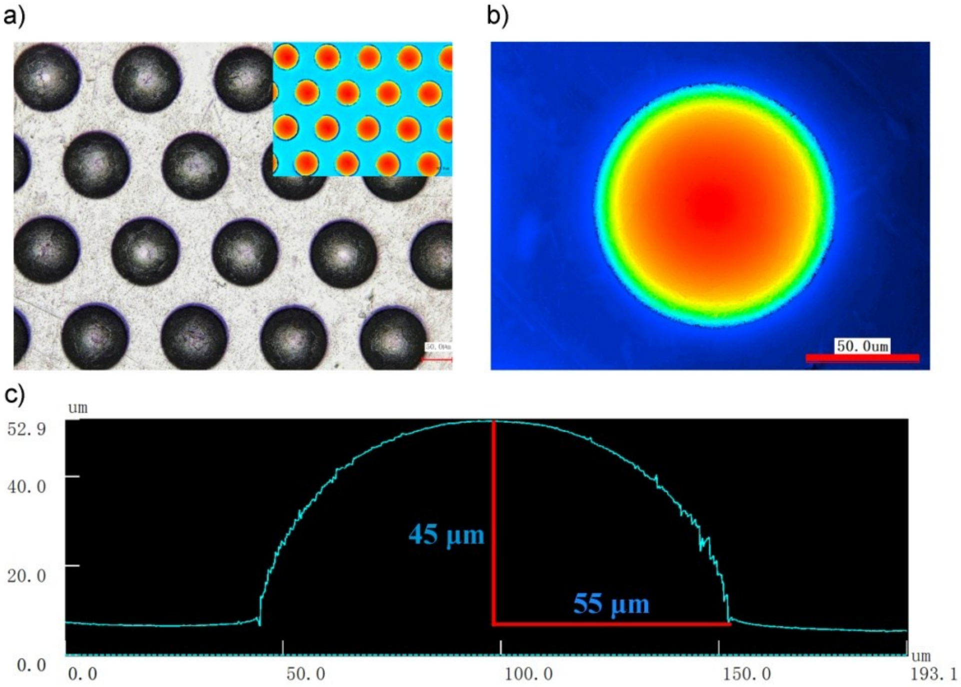

Figure S2. The characterization of mold electrode. a) The Confocal laser microscope image showing the convex microlens array, inset is the height image. b) The 3D height image of a single microlens. c) The topography profile of b).

3. The AFM characterization of hierarchical nanorings

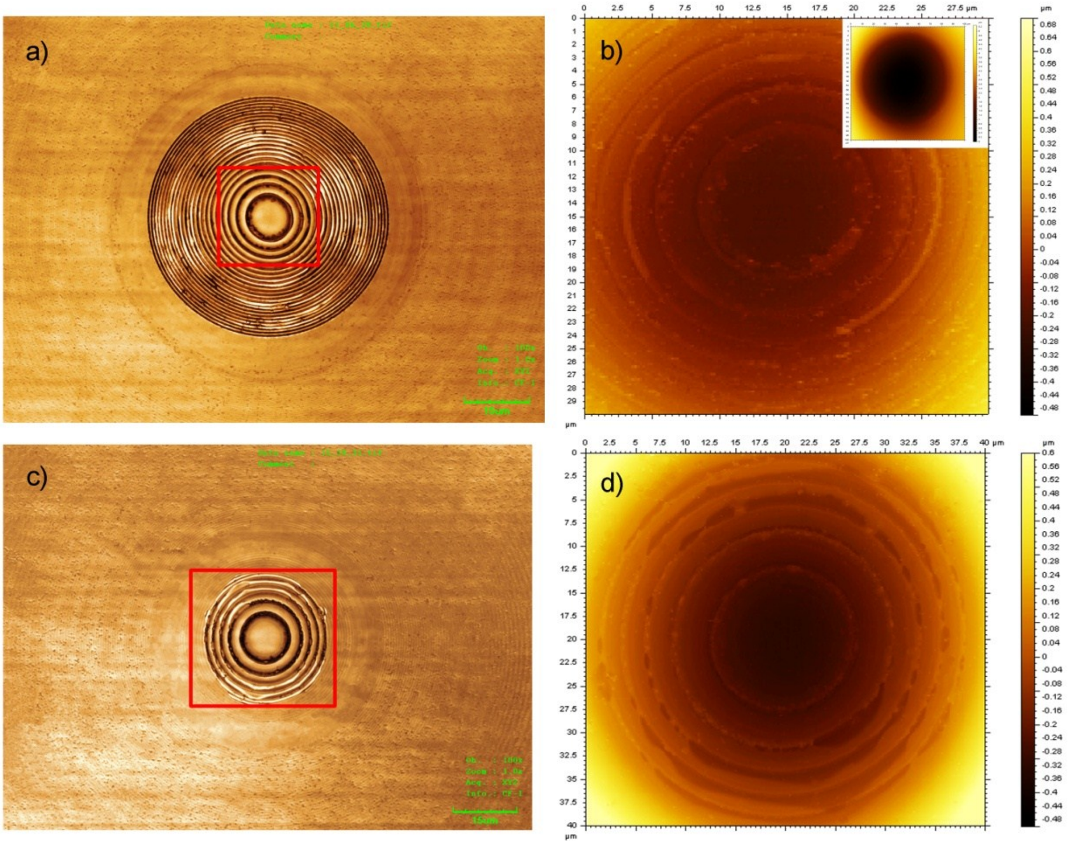

Figure S3. The AFM characterization of hierarchical nanorings. a), c) The confocal laser microscopic images of the hierarchical nanorings fabricated at 20mN and 5 mN respectively. b), d) The AFM images of the marked central area of a) and c), respectively. The inset in b) shows the AFM image of a) with large area.

4. Control experiments without contact force

To figure out whether the hierarchical concentric nanorings are generated through buckling effect, control experiments in which the $Ga_xIn_{1-x}P$ workpiece does not contact with mold electrode was performed. The stepper motor combined with piezo motor is employed to accurately control the distance between workpiece and mold electrode (500 nm). The mold electrode was biased at 1.0 V (vs. SCE) for 1 hour to generate the etchant bromine. L-cystine was also added to ensure the resolution of CELT. The concave microlens fabricated under these conditions (Figure S4) are almost complementary with the mold electrode. The results indicate the compression between mold electrode and workpiece is essential to generate the buckling nanorings.

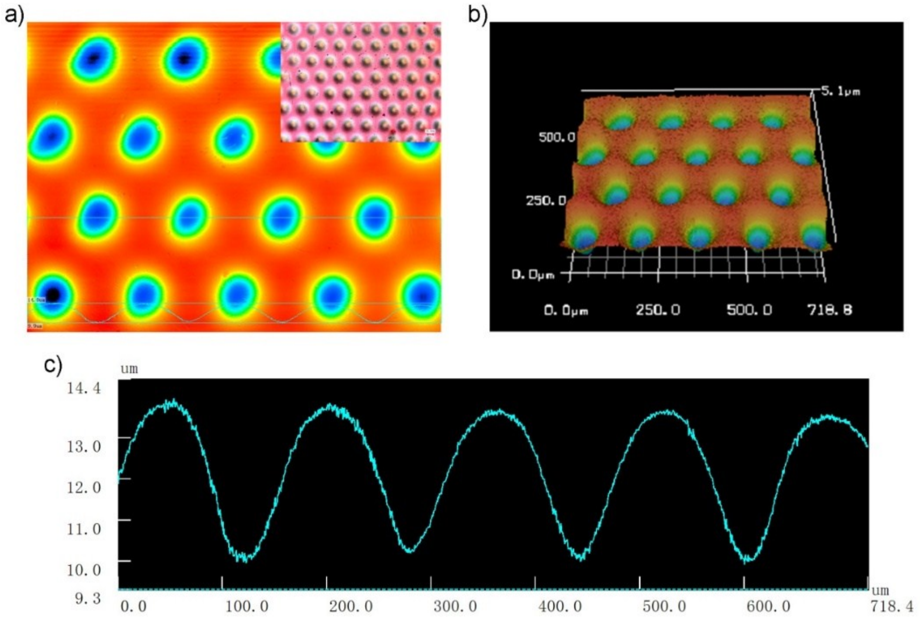

Figure S4. The characterization of concave microlens fabricated through CELT without compression between $Ga_xIn_{1-x}P$ workpiece and mold electrode. a) The Confocal laser microscope image of concave microlens. Inset is image with large area. b) The 3D image of a). c) The topography profile of b).

5. Tuning the hierarchical nanorings through contact force

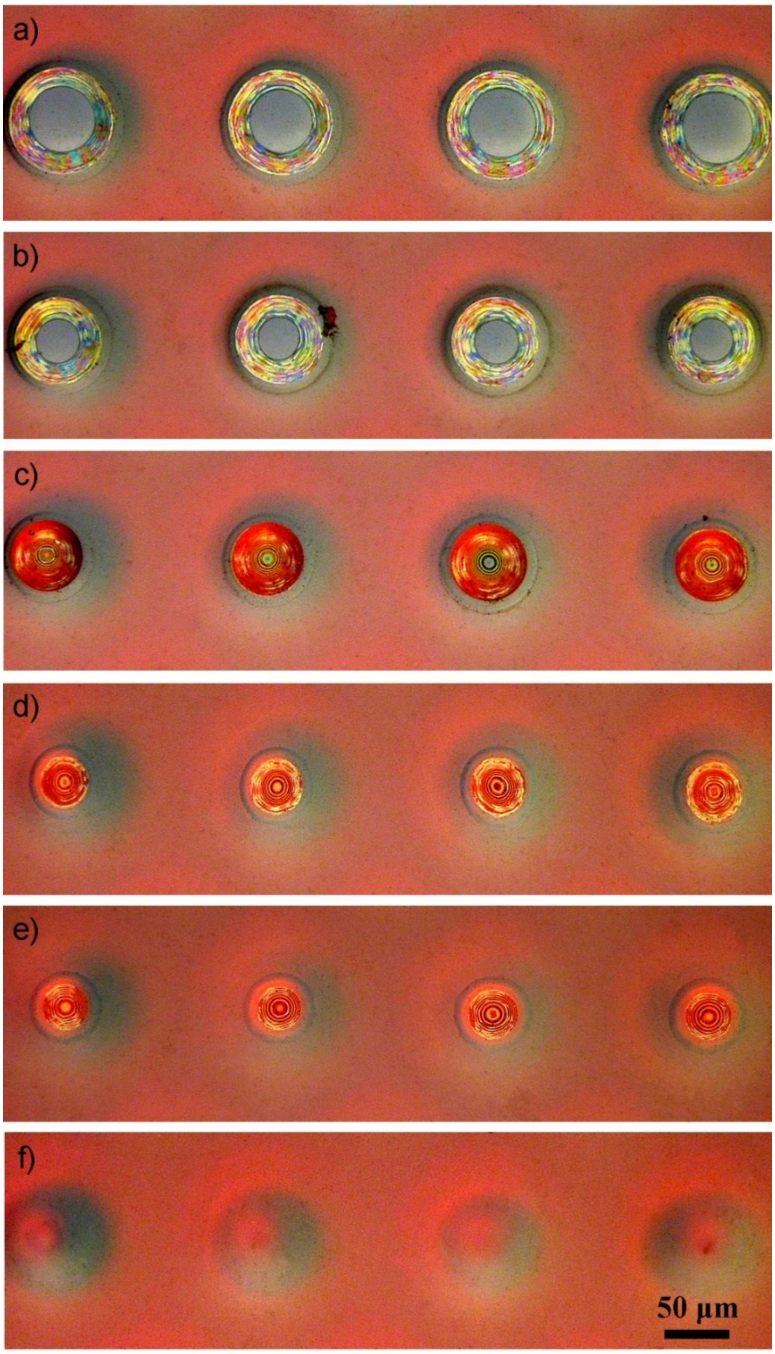

Figure S5. Tuning the hierarchical nanorings through contact force. The contact force decreases gradually from a) to f): 80 mN, 60 mN, 20 mN, 10 mN, 5 mN, 0 mN.

6. FEM simulation of buckling behavior

6.1 The reversible buckling process

In order to research the buckling behavior on continuously curved surface, the finite element method was adopted to simulate the three dimensional buckling phenomena on PMMA convex mirolens covered with platinum film.[3] Considering an elastic medium that occupies a domain Ω with boundary Γ, the variational equation of static equilibrium is:

$$\delta W^{int} - \delta W^{ext} = 0, \delta W^{int} = \int_{\Omega} \delta E_{ij} S_{ij} d\Omega, \delta W^{ext} = \int_{\Gamma^t} \delta u_i t_i d\Gamma \qquad \text{(S4)}$$

where δ is variational symbol, δW^{int} and δW^{ext} are the internal and external virtual work. E_{ij} is the Green-Lagrangian strain and S_{ij} is the second Piola-Kirchhoff stress. u_i is the displacement and t_i denotes the applied traction on the natural boundary Γ^t. The value of subscripts *i* and *j* range from 1 to 3. The Saint-Venant type of constitutive equation (Equation 5 in the main text) is employed to relate the stress and strain tensors.

Introducing the finite element approximation into Equation S4 and performing standard linearization for the internal virtual work gives:

$$\Delta \delta W^{\text{int}} = \delta \mathbf{d}^T (\mathbf{K}^M + \mathbf{K}^G) \Delta \mathbf{d} \qquad \text{(S5)}$$

where Δ is the incremental operator, and $\mathbf{d}$ is the displacement vector. $\mathbf{K}^M$ and $\mathbf{K}^G$ are the material and geometric stiffness matrices, respectively. The buckling analysis refers to the following eigenvalue problem:

$$(\mathbf{K}^M - \beta \mathbf{K}^G) \Delta \mathbf{d} = \mathbf{0} \qquad \text{(S6)}$$

where β is the buckling load and $\Delta \mathbf{d}$ is the corresponding buckling mode to be determined. It is noted that the buckling mode denote the shape of the buckling specimen.

6.2 Details of FEM simulation

The simulation was carried out using the finite-element package ANSYS with 2-node axisymmetric shell element and 8-node axisymmetric quadrilateral elements (Shell51, Plane82). The shell elements have four degrees of freedom at each node. The 8-node quadrilateral elements have two degrees of freedom per node, which are well suited to model curved boundaries. These nodes are merged to simulate the strong adhesion between Pt film

and PMMA substrate. The Pt film and PMMA substrate are partitioned by 562 two-node axisymmetric shell elements and 158365 eight-node axisymmetric quadrilateral elements. Note that the fineness of discretization is required by the nano-scale buckling. The eigenvalue buckling analysis is performed to obtain the possible buckling modes of the structure subjected to certain boundary conditions and loading. The loading is typically defined by the applied force or the displacement at the contact area of the structure. The eigenvalues and eigenvectors of the tangent stiffness matrix correspond to the magnitude of load and shape of the possible buckling modes of the structure. The Lanczos iteration method is used herein to compute the eigenmodes. Once a set of eigenmodes are chosen, geometrical imperfections are introduced in the form of out-of-plane deformations of the membrane. A geometrically nonlinear incremental analysis is carried out using the Newton–Raphson solution method with arc-length control. Then the converged buckling behavior can be achieved.

6.3 Tuning the nanorings through the distribution of stress and thickness of platinum film

Figure S6 shows the distribution of the third principal stresses in the non-uniform Pt film for four different loading cases. The compressive stress is decreased from top to the bottom. The increasing contact force yields larger third principal stress, which leads to denser buckling nanorings. This is consistent with the buckling behavior of planar film suffering uniaxial stress (Equation S7 and Equation S8).[4]

$$\lambda_0 = 2\pi h_f \left(\frac{\bar{E}_f}{3\bar{E}_s} \right)^{1/3} \tag{S7}$$

$$\lambda = \frac{\lambda_0}{(1+\varepsilon_{pre})(1+\xi)^{1/3}}, \quad \xi = \frac{5\varepsilon_{pre}(1+\varepsilon_{pre})}{32} \tag{S8}$$

where λ_0 defined by equation S7 is the wavelength only applicable in small deformation.[5] λ defined by equation S8 is applicable in both small and large deformation.[4] h_f is the thickness of plane film. $\bar{E}_f = E_f / (1-\nu_f^2)$, $\bar{E}_s = E_s / (1-\nu_s^2)$, E_f and ν_f and E_s and ν_s are the Young's modulus and Poisson's ratios of plane film and elastic substrate, respectively. ε_{pre} is the prestrain of elastic substrate.

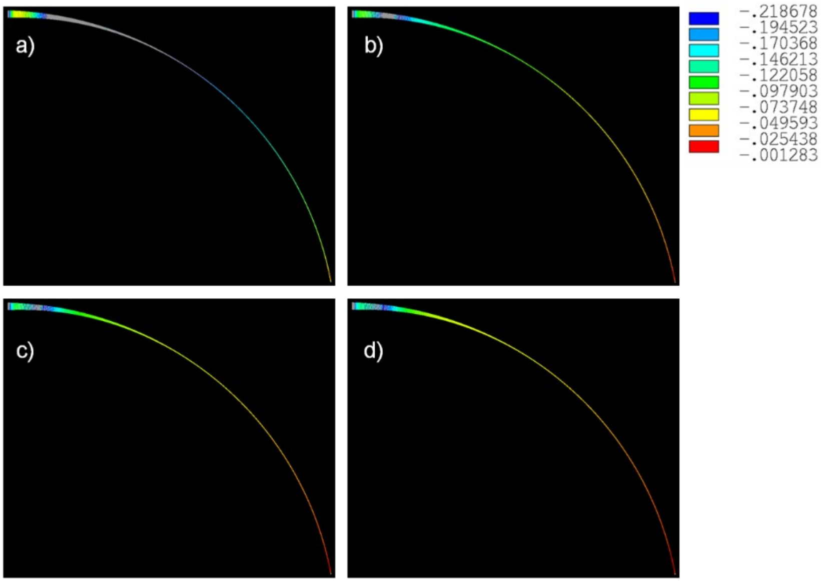

Figure S6. FEM simulation results of the stress distribution at convex microlens mold electrode with different contact force: a) 60 mN. b) 40 mN. c) 20 mN. d) 10 mN. The thickness of platinum film changes from 248 nm at the top to 30 nm at the bottom.

Meanwhile the space between nanorings is also dependent on the bending rigidity of Pt film and the rigidity of elastic substrate (Equation S9).[6]

$$\lambda \sim \left(\frac{B}{K}\right)^{1/4}, \quad B = E_f h_f^3 \Big/ \left[12\left(1-\nu_f^2\right)\right] \tag{S9}$$

where B and K is the bending stiffness of thin film and elastic substrate, respectively. The bending rigidity of Pt film (B) is related to its thickness (h_f). So thicker Pt film has larger bending rigidity and tends to produce sparser nanorings. Thus the bending rigidity and the stress state herein play opposite roles. For the convex microlens, the bending rigidity of Pt film dominates the space between nanorings. For uniform thickness with 90 nm, the stress distribution is similar to that of the non-uniform case. However, the bending rigidity of Pt film is uniform. So the distribution of stress dominates the space between nanorings, which leads to the increasing space between nanorings from center to outside (Figure S7). We also considered another situation in which the Pt thickness changes from 300 nm at the top to 80 nm at the bottom. The loading conditions are the same as previous examples. In this case, the influence of bending rigidity and stress state is well-balanced. The space between nanorings tends to become more uniform (Figure S8).

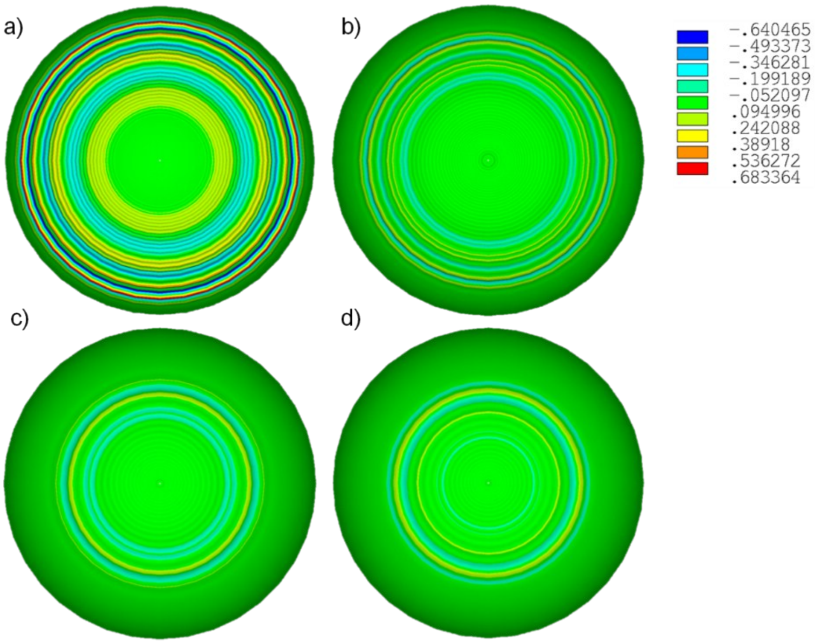

Figure S7. FEM simulation results of buckling behavior at convex microlens mold electrode with different contact force: a) 60 mN. b) 40 mN. c) 20mN. d) 10mN. The thickness of platinum film is uniform with 90 nm.

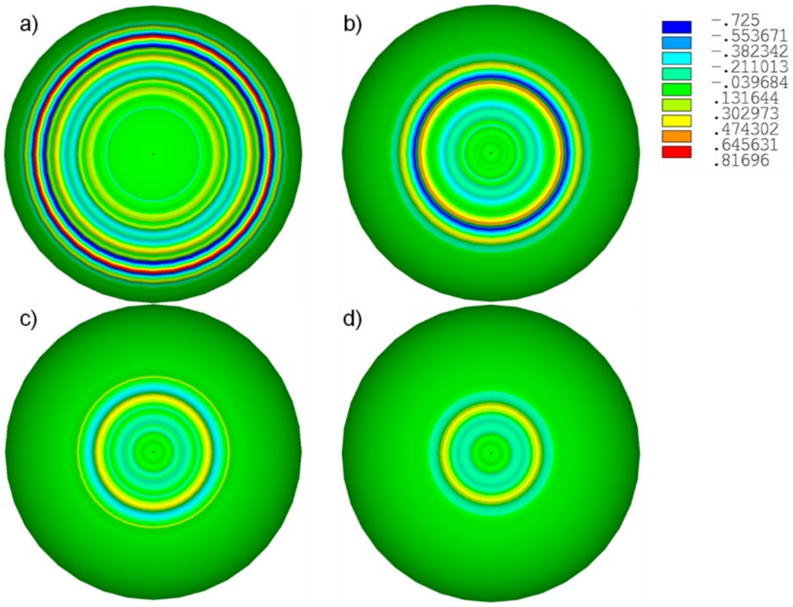

Figure S8. FEM simulation results of buckling behavior at convex microlens mold electrode with different contact force: a) 60 mN. b) 40 mN. c) 20mN. d), 10mN. The thickness of platinum film changes from 300 nm at the top to 80 nm at the bottom.

7. The photoluminescence images of hierarchical nanorings

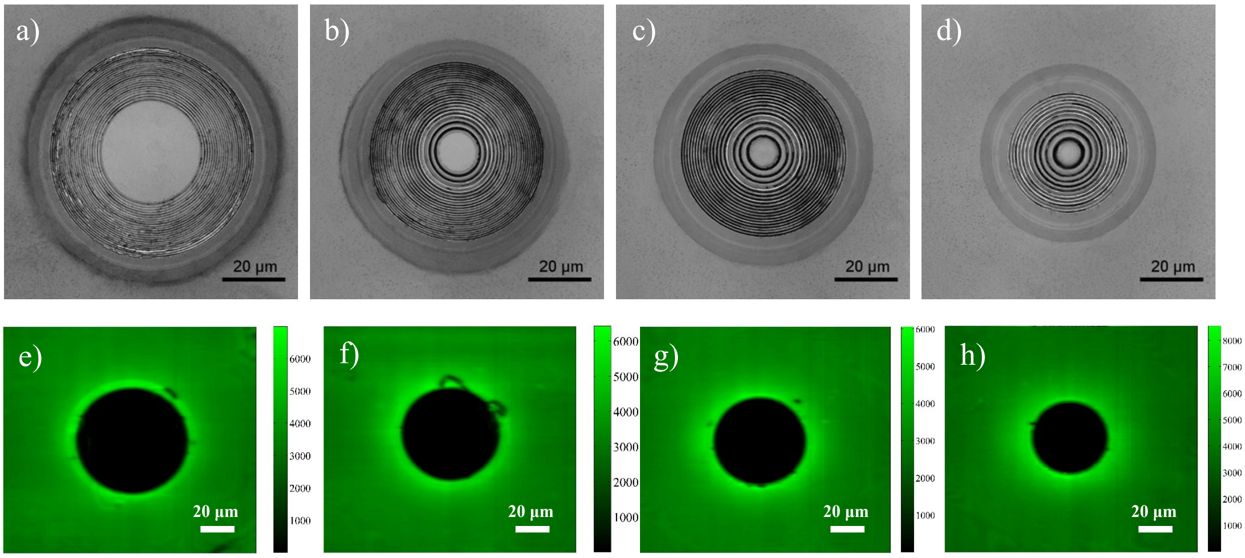

Figure S9. The photoluminescence images of the hierarchical concentric nanorings. a) to d) The confocal laser image. e) to h) The corresponding Raman mapping of the PL band of $Ga_xIn_{1-x}P$ at 630 nm.

8. Tuning the hierarchical nanogrooves through contact force

If the microstructure is a convex hemi-cylinder, a concave hemi-cylinder with hierarchical nanogrooves will be obtained (Figure S10a). We also can tune the hierarchical nanogrooves through contact force like the hierarchical nanorings discussed above. The width and space of nanogrooves also decrease with the increase of contact force. Figure S10b and Figure S10d are the SEM images of nanogrooves fabricated at 60 mN and 10 mN, respectively. The width of central nanogrooves at 10 mN and 60 mN is 1.3 μm and 350 nm, respectively. The space of the central nanogrooves at 10 mN and 60 mN is 2.6 μm and 800 nm, respectively. The width and space of nanogrooves also decrease from the center of concave hemi-cylinder to outside (Figure S10d). The nanogrooves also trends to become more uniform with increasing contact force (Figure S10b).

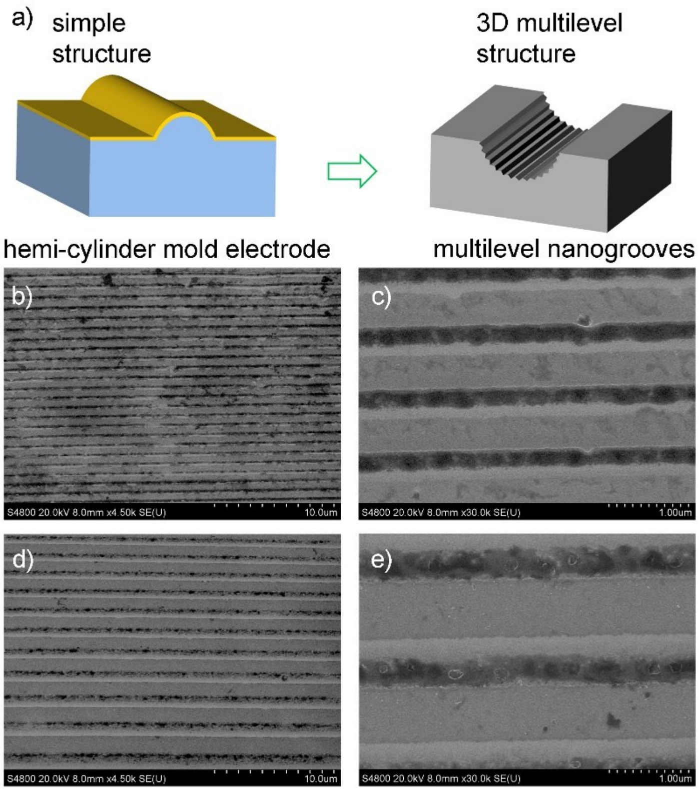

Figure S10. Hierarchical nanogrooves fabricated by ECBM. a) Schematic graph of ECBM using a convex hemi-cylinder mold electrode. b) and d) The SEM images of nanogrooves fabricated at 60 mN and 10 mN. c) and e) The localized SEM images of b) and d).

9. The characterization of Young's modulus of PMMA substrate

In general, polymer itself has a small Young's modulus. However, in the practical applications, it is important to improve the mechanical strength of polymer materials through tuning the orientation degree, molecular weight, grafting and modification. *The Young's modulus of commercially available PMMA materials ranges from 3 to 30 GPa.* To figure out the Young's modulus of the PMMA mold, experiments are performed by an electronic universal testing machines with extensometer (AGS-X, Shimadzu, Japan). Figure R1 shows the stress-strain graph of PMMA. The stress (σ) on the section of the dumbbell shaped PMMA board is described by:

$$\sigma = \frac{P}{bd} \qquad \text{(S10)}$$

where P is the load, b (1.95 mm) is the wide and d is the thickness (9.83 mm) at the middle part of dumbbell shaped PMMA. The strain (ε) is the relative deformation of the PMMA which is described by

$$\varepsilon = \frac{L - L_0}{L_0} \tag{S11}$$

Where L and L_0 is the length of the PMMA boards after and before stretch. The Young's modulus is defined by

$$E = \frac{\sigma}{\varepsilon} \tag{S12}$$

Fitting the initial straight line segment through the origin, the Young's modulus is obtained and showed in Table R1. The average value of Young's modulus of the PMMA is 16.7 Gpa. For the convenience of FEM simulation, we use a rounding value of 20 Gpa. Actually, as shown in Equation S7, the period of the wrinkles is proportional to the thickness of film (h_f) and the cube root of $\bar{E}_f / (3\bar{E}_s)$. The rounding number (20 GPa) only introduce a difference less than 6%. So we think it is reasonable to use the rounding number (20 GPa) as the Young's modulus of PMMA substrate. We add this part in the supporting information (See S9).

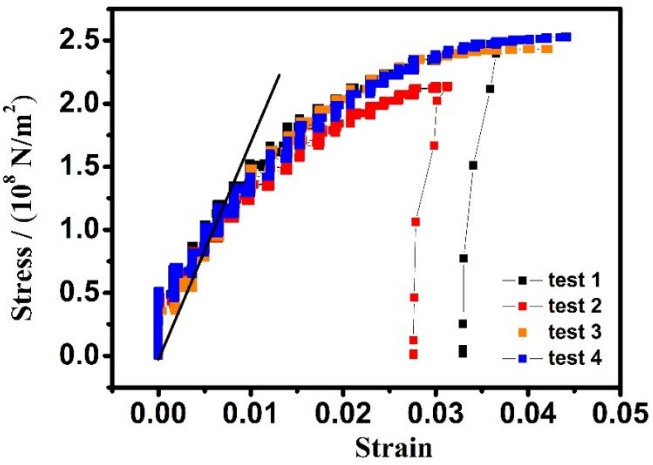

Figure S11. The stress-strain curves of PMMA in the tensile test

Table S1. The Young's modulus of the PMMA

Sample	Test-1	Test-2	Test-3	Test-4

E(Gpa)	17.7	17.6	16.1	15.5

10. The non-uniform distribution of Pt film on PMMA mold

Since the microlens is a hemisphere with a height of 45 μm, it is difficult to be imaged by AFM and STM scrapping mode. Actually, we tried a SEM experiment by the cutting method to expose the cross section of the microlens. Unfortunately, the Pt film is destroyed. From the SEM image, it is observed that the thickness of Pt film is not uniform from top to bottom. When observing the top area, the thin film get melted and difficult to be imaged because of the highly focused electron beam. Nevertheless, the situation of bottom area is a little bit better due to the energy can be transported to the adjacent area. It can be observed faintly that the thickness is about 30 nm at the bottom as well as at about 250 nm at the bottom. That is why we consider it changes gradually from 248 nm at the top to 30 nm at the bottom.

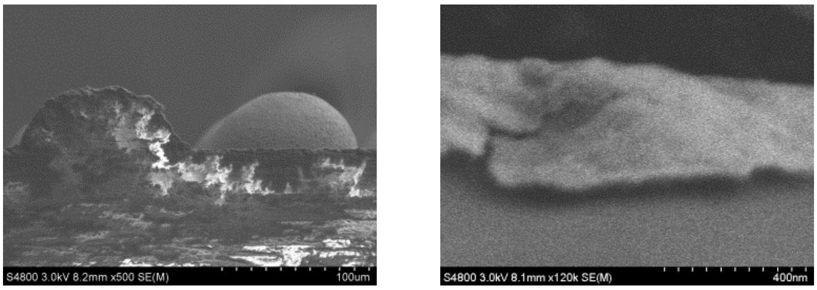

Figure S12: (left): the SEM image of the cross section of microlens; (right) the SEM image of Pt film at the bottom.

11. AFM characterization of the amplitude of concentric nanorings

From the 3D confocal laser microscopy image of the Fresnel nanorings fabricated at 20 mN contact force (Figure 2c in the main text), we can know that the center nanoring has an amplitude of almost 50 nm and decays from center to outside. We further characterized the amplitude of nanoring fabricated at different contact force by atomic force microscopy (Figure S12). The amplitude of the first five nanorings fabricated at 20 mN contact force (Figure S12c) is 34, 32, 22, 15 and 13 nm. While the amplitude of the nanorings fabricated at 60 mN contact force (Figure S12f) is almost 50 nm. The downtrend of the amplitude at 60 mN is less obvious than that at 20 mN. Just as the stress distribution we discussed above, according to the relationship between the prestrain and amplitude described by Equation S13, the Pt film on the top suffering larger compressive stress and trends to produce wrinkles with

larger amplitude. On the contrary, the Pt film on the bottom suffering smaller compressive stress and trends to produce wrinkles with smaller amplitude. In hence, the amplitude of the nanorings is decreased from the center to outside, and the amplitude of all the nanorings fabricated at 60 mN contact force is larger than that at 20 mN. Meanwhile, the decreased amplitude of nanorings from the center to outside also should attributed to the non-uniform thickness of Pt film (Equation S13). The Pt film on the top having thicker thickness and trends to produce wrinkles with larger amplitude. On the contrary, the Pt film on the bottom having thinner thickness and trends to produce wrinkles with smaller amplitude.

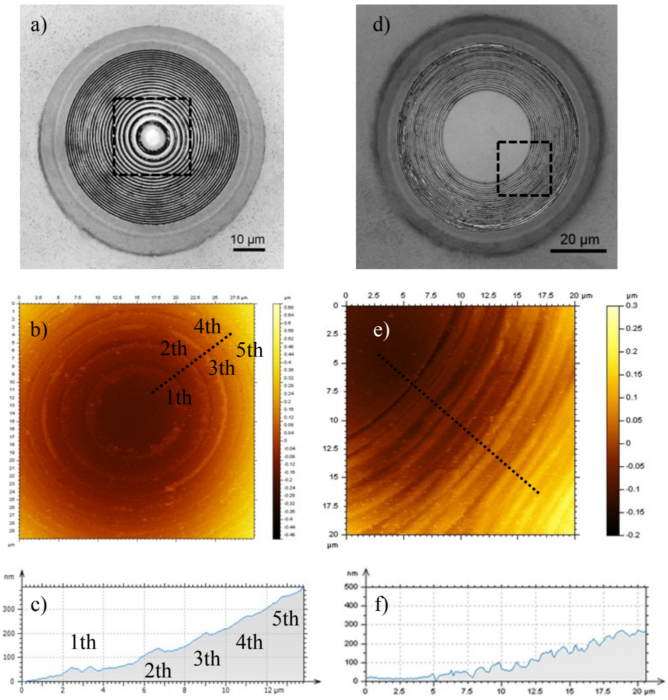

Figure S13 The AFM characterization of hierarchical nanorings. a) and d) are the confocal laser microscopy images of the Fresnel nanorings fabricated at 20 mN and 60 mN contact force, respectively. b) and e) are the AFM images of the locations outlined by the dotted line squares in a) and d), respectively. c) and f) are the cross-sectional profiles of the locations outlined by the dotted line in b) and e), respectively.

References:

[1] Z. W. Tian, Z. D. Fen, Z. Q. Tian, X. D. Zhuo, J. Q. Mu, C. Z. Li, H. S. Lin, B. Ren, Z. X. Xie, W. L. Hu, *Faraday Discuss.* 1992, **94**, 37-44.

[2] L. Zhang, X. Z. Ma, J. Tang, D. S. Qu, Q. Y. Ding, L. N. Sun, *Electrochim. Acta* 2006, **52**, 630-635.

[3] O. C. Zienkiewicz and R. L. Taylor, The Finite Element Method For Solid And Structural Mechanics, Butterworth-Heinemann, Oxford, 6 edn., 2005.

[4] H. Jiang, D. Y. Khang, J. Song, Y. Sun, Y. Huang, J. A. Rogers, *Proc. Natl. Acad. Sci. U. S. A.* 2007, **104**, 15607-15612.

[5] a) A. L. Volynskii, S. Bazhenov, O. V. Lebedeva, N. F. Bakeev, *J. Mater. Sci.* 2000, **35**, 547-554; b) J. Y. Chung, A. J. Nolte, C. M. Stafford, *Adv. Mater.* 2011, **23**, 349-368.

[6] E. Cerda, L. Mahadevan, *Phys. Rev. Lett.* 2003, **90**, 074302.
